# Death from Chloroform

**Published:** 1859-08

**Authors:** 


### DEATH FROM CHLOROFORM.

On Tuesday last, another death from chloroform occurred at the Royal Opthalmic Hospital. The patient, a healthy looking girl of fifteen, was about to be operated on for strabismus. She inhaled the anæsthetic without any unusual symptoms, when she uttered a sudden and very peculiar shriek. After this, she soon became quite insensible; the speculum was introduced, and the operator commenced. The chloroform had been administered on a piece of folded linen, and the time and quantity consumed in the inhalation had both been about the average (neither had been measured.) Soon after the first snip with the scissors, the operator's attention was called to the lividity of the girl's countenance, and on the finger being applied to the wrist, she was found to be quite pulseless. Mr. Critchett had already forced open her mouth, and dragged the tongue forwards. Artificial respiration was at once commenced, and, without any intermission, was kept up for about an hour. For a long time sanguine hopes were entertained that she would recover, as slight gasping efforts at inspiration continued to occur at intervals. The pulse, however, never returned, and at length it became apparent that she was quite dead. Movements of of the nostrils, as if in the attempt to inspire, were apparent for at least half an hour after the pulse had ceased. A brandy enema was given almost immediately after the first symptoms occurred, and it was then found that the sphincter ani was quite relaxed. Mr. Critchett, Mr. Bowman, Mr. Hulke, and a large staff of assistants were present, and everything that was thought desirable in the way of treatment was effected with the utmost efficiency and promptitude. The following day a post mortem examination took place. Dr. Baden, who conducted it, informs us that the most noticeable lesion met with was the presence of air, in considerable quantity, in the right chambers of the heart. The left side of the heart was empty, but the right contained a small quantity of spumous and fluid blood, with probably not less than two ounces of gas. In the lungs were numerous spots of apoplectic extravasation, probably by the very efficient manner in which artificial respiration had been kept up. There was no organic disease in the body. The uterine organs showed that menstruation (for the first time) was just about to be established. —*Med. Times and Gaz., June* 4.

Two Cases of Death from Chloroform.—Two deaths have occurred from chloroform in the hospitals of Paris, in the hands of two surgeons, not less eminent for their knowledge than their prudence, and the precautions they are accustomed to employ in the administration of this agent. The first case was a man aged 44, strong and vigorous. It was proposed to reduce a luxation of the shoulder. From fifteen to twenty grammes of the anæsthetic were employed. The chloroformization followed its usual course: fi st *excitation*, then *relaxation*. The pulse continued calm, as well as the breathing; the expression of the countenance was not changed; nothing indicated an unfavorable result. Relaxation having been obtained, Ritchet proceeded with the reduction, having removed the chloroform from the patient. Suddenly, the reduction being accomplished, the pulse ceased on both sides; there were no longer præcordial palpitations, although respiration continued. This soon ceased; then Ritchet used artificial respiration. Three inspirations were obtained, but the patient succumbed, without any movements of the heart being reëstablished.

The second case was a girl, aged seven and a half years, in the service of M. Marjolin. Four grammes of chloroform were employed. It was employed as in the preceding case, in a bloodless operation—that for coxalgia (Bonnet's treatment.) The child submitted readily to the inhalations. The first attempt was followed by cries from the child, who endeavored to place its free hand on the distant hip. In a few seconds the anæsthetic sleep and relaxation were produced, although the anæsthesia was not profound, since but few manipulations had been made, when the cries and agitation were reëstablished. The surgeon ceased his operations, and suddenly the cries and muscular resistance ceased. Instinctively, and as if from a sad presentiment, Majorlin looked at the child. The physiognomy was strange; the head was turned towards the bolster; the countenance more highly colored than a few moments before; eyes fixed and half closed. The pulse and heart being examined, no pulsations were perceived; three or four inspirations, becoming weaker and weaker, then took place; all relief proved useless; death was certain. In these two cases the pulse and the heart ceased to furnish palpitations, although the respiration continued. There was no asphyxia, but a contrary effect in each case. In both, the countenance, at the moment of death, preserved its color; and hence death did not take place from syncope. How, then, did the chloroform act? Was it by direct action on the heart and lungs, which were as if paralyzed by it? These questions have not as yet been settled.—*Jour. de Pharm.*, *and Am. Med. Monthly.*

